# Network-based identification of critical regulators as putative drivers of human cleft lip

**DOI:** 10.1186/s12920-018-0458-3

**Published:** 2019-01-31

**Authors:** Aimin Li, Guimin Qin, Akiko Suzuki, Mona Gajera, Junichi Iwata, Peilin Jia, Zhongming Zhao

**Affiliations:** 1 Shaanxi Key Laboratory for Network Computing and Security Technology, School of Computer Science and Engineering, Xi’an University of Technology, Xi’an, 710048 Shaanxi China; 2 Center for Precision Health, School of Biomedical Informatics, The University of Texas Health Science Center at Houston, 7000 Fannin St., Suite 820, Houston, TX 77030 USA; 3 School of Software, Xidian University, Xi’an, 710071 Shaanxi China; 4 Department of Diagnostic and Biomedical Sciences, School of Dentistry, The University of Texas Health Science Center at Houston, Houston, TX 77054 USA; 5 Center for Craniofacial Research, The University of Texas Health Science Center at Houston, Houston, TX 77054 USA; 6 MD Anderson Cancer Center UTHealth Graduate School of Biomedical Sciences, Houston, TX 77030 USA

**Keywords:** Cleft lip, Feed forward loop, Regulatory network, Disease-causing gene, microRNA, Transcription factor

## Abstract

**Background:**

Cleft lip (CL) is one of the most common congenital birth defects with complex etiology. While genome-wide association studies (GWAS) have made significant advances in our understanding of mutations and their related genes with potential involvement in the etiology of CL, it remains unknown how these genes are functionally regulated and interact with each other in lip development. Currently, identifying the disease-causing genes in human CL is urgently needed. So far, the causative CL genes have been largely undiscovered, making it challenging to design experiments to validate the functional influence of the mutations identified from large genomic studies such as CL GWAS.

**Results:**

Transcription factors (TFs) and microRNAs (miRNAs) are two important regulators in cellular system. In this study, we aimed to investigate the genetic interactions among TFs, miRNAs and the CL genes curated from the previous studies. We constructed miRNA-TF co-regulatory networks, from which the critical regulators as putative drivers in CL were examined. Based on the constructed networks, we identified ten critical hub genes with prior evidence in CL. Furthermore, the analysis of partitioned regulatory modules highlighted a number of biological processes involved in the pathology of CL, including a novel pathway “Signaling pathway regulating pluripotency of stem cells”. Our subnetwork analysis pinpointed two candidate miRNAs, *hsa-mir-27b* and *hsa-mir-497*, activating the Wnt pathway that was associated with CL. Our results were supported by an independent gene expression dataset in CL.

**Conclusions:**

This study represents the first regulatory network analysis of CL genes. Our work presents a global view of the CL regulatory network and a novel approach on investigating critical miRNAs, TFs and genes via combinatory regulatory networks in craniofacial development. The top genes and miRNAs will be important candidates for future experimental validation of their functions in CL.

**Electronic supplementary material:**

The online version of this article (10.1186/s12920-018-0458-3) contains supplementary material, which is available to authorized users.

## Background

Cleft lip (CL) is one of the most common congenital birth defects in humans, affecting approximately 1/700 live births [1]. Specifically, CL is characterized by unilateral or bilateral clefts in the upper lip resulting from failure of the growth and/or fusion of the nasal and maxillary processes during craniofacial development. CL is a multifactorial disorder, caused by a combination of genetics and environmental factors and influences feeding, speech, appearance, and more [2]. To dissect and explain complex characteristics of CL, a deep understanding of its fundamental genetics is crucial. During the recent decade, numerous genetic studies (e.g. genome-wide association studies (GWAS), copy number variation, mRNA and miRNA expression profiling, chromatin modifier-associated region identification assay, enhancer reporter gene assay and methylation) have been performed, which substantially contributed to the discovery of human CL genes and their potential functions [3–7]. Previous studies have suggested that mutations and variations in the *IRF6*, *p63*, and *TGFA* genes were etiologic in CL [1, 8, 9]. Some studies indicate that p63 protein activates *IRF6* transcription through binding its enhancer [10, 11]. p63 *and* IRF6 cooperate within a feedback regulatory loop in order to determine the fate of epithelial cells in proliferation vs. differentiation during palate development [12]. Disruption of this loop by mutations in either IRF6 *or* p63 might increase the susceptibility to CL. Although these results are still limited to unveil a systematic view of the biological process of CL, it provides us a clue that analysis of gene regulation relationships would be powerful for identification of critical genes or regulatory motifs that drive CL. This is also consistent with that craniofacial development is often involved in many biological processes and cascades, which can be detected at the molecular levels (e.g., gene expression, enhancer, transcription factor, and post-transcriptional regulation).

MicroRNAs (miRNAs) and transcription factors (TFs) are key regulators of gene expression. miRNAs are small non-coding RNAs (composing about 21~22 nucleotides) that regulate gene expression at the post-transcriptional level. In animals, a mature miRNA typically binds to the 3′ untranslated regions (3’UTRs) of the target mRNAs, and consequently leads to degradation and translational repression of the mRNAs [13]. Previous studies have revealed that overexpression of *miR-140* could result in CL in zebrafish [14], and that a single nucleotide polymorphism (SNP) located in *pre-miR-140* was found to be associated with cleft palate (CP) in humans [15]. Recently, hundreds of miRNAs are reported to have aberrant expression in CL [16]; however, researchers have yet to find out which miRNAs play prominent roles in the pathological process of CL or the interrelated targets of these miRNAs.

Gene transcription is typically regulated by TFs in cellular systems. TFs control the rate of transcription from DNA to mRNAs by binding to the transcription factor binding sites (TFBS) in the promoter regions of the target genes [17]. miRNAs and TFs can be co-regulated or regulated with each other by several scenarios: miRNAs’ expression may be regulated by TFs [18], TFs and miRNAs may mutually regulate one another to represent feedback loops (FBLs), or alternatively, both TFs and miRNAs may simultaneously regulate their joint target genes and form feed-forward loops (FFLs). Network analysis, including motifs such as FBLs and FFLs, is an effective way to explore the fundamental global topological structures of molecular networks [19]. For example, miRNA-TF co-regulation is one of the simplest but important FFL types. So far, miRNA-TF co-regulation network analyses have helped investigators identify important regulatory motifs and understand cellular regulatory mechanisms in several diseases [19]. In gene regulatory networks, typical FFL motifs are composed of three nodes: miRNA, TF, and their jointly regulated target gene. Recently, FFL-based mixed regulatory networks have served as promising tools to elucidate complex diseases, such as schizophrenia [20], glioblastoma multiforme [21], ovarian cancer [22], non-small cell lung cancer [23], colorectal cancer [19], and osteosarcoma [24].

In this study, we employed a regulatory network-based approach for systematically investigating gene regulation patterns in CL. Using the CL candidate genes that we systematically collected and curated, we first examined four types of regulatory pairs: miRNA-gene, miRNA-TF, TF-gene and TF-miRNA. Based on these interaction pairs, we obtained three types of FFLs and named them motifs A, B, and C. Then, we used these motifs to construct four networks (motif A network, motif B network, motif C network, and combined network of motifs A, B, and C). We further evaluated the functional features of genes in these networks using the pathway enrichment analysis based on the Gene Ontology (GO) and the Kyoto Encyclopedia of Genes and Genomes database (KEGG) pathway annotations. We further used an independent gene expression data set (GEO accession number: GSE7759) to verify the findings from the combined network. This dataset could verify 64.8% of TF-gene edges. We extracted ten hub genes and identified three modules from the combined network for functional enrichment analysis. Notably, by investigating the network modules, we found the signaling pathway that regulate pluripotency of stem cells was significantly involved in CL; such finding has not been reported before. Our network analysis also highlighted a CL subnetwork that is related to Wnt pathway, and pinpointed five miRNAs, *hsa-mir-374b, hsa-mir-381, hsa-mir-374, hsa-mir-27b* and *hsa-mir-497*, involved in it. Through a gene expression and network topological analysis, we identified two of these miRNAs, *hsa-mir-27b* and *hsa-mir-497*, were essential for the activation of the Wnt pathway. Previous studies reported that both of these miRNAs were involved in the etiology of CL. Thus, our miRNA-TF-mediated regulatory network analysis was consistent with previous studies, suggesting the efficacy of the approach. Taken together, our integrated miRNA-TF mediated regulatory networks are useful for detecting genes, regulators and networks, which provides insights into the regulation of CL. Future experimental studies of these top candidates and regulations will help advance our knowledge in CL biology and the CL etiology.

## Results

### Integrative framework for identification of critical disease-causing genes based on networks

**Fig. 1:**
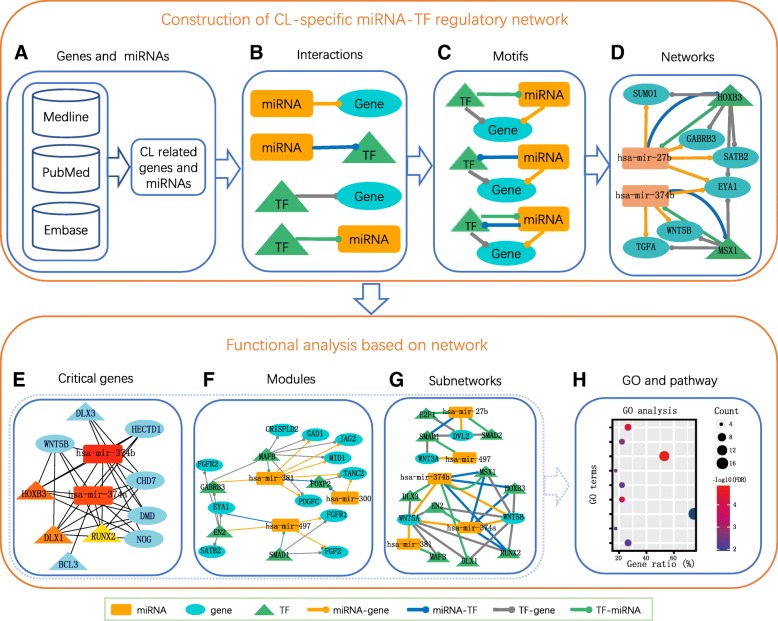
Schematic flowchart for constructing and analyzing the comprehensive miRNA–TF co-regulatory networks in cleft lip (CL). (**a**) CL-related genes were collected and manually curated from main biomedical databases including Medline, PubMed and Embase. The miRNAs targeting these genes were identified from four miRNA-target interaction databases: miRanda, PITA, TargetScan and miRTarBase. (**b**) Four types of regulation relationships: TF-gene, TF-miRNA, miRNA-TF and miRNA-gene. (**c**) Three regulatory motifs measured by feed forward loop (FFL). (**d**) Construction of the miRNA–TF co-regulatory network. The combined network of all FFLs consisted of 8 miRNAs, 15 TFs, 26 genes and 163 edges. (**e**) Identification of critical genes (TFs, miRNAs and genes) and analysis of their potential functions. (**f**) Identification of network modules and analysis of their potential functions. (**g**) Extracting miRNA–TF co-regulatory subnetworks based on a specific pathway (Wnt pathway). (**h**) GO and pathway enrichment analysis. Blue ellipse: gene; orange rectangle: miRNA; green triangle: TF; blue line: miRNA-TF; orange line: miRNA-gene; green line: TF-miRNA; gray line: TF-gene

Figure 1 elaborates our combinatory framework to construct comprehensive miRNA–TF co-regulatory networks and sort out critical genes and modules in CL. These networks were composed of FFL regulations among three main components: miRNAs, TFs and genes. In this study, we used the genes and miRNAs that we searched from several literature databases followed by careful, manual curation for human CL (Additional file 1). Specifically, through an extensive exploration of three major biomedical databases, Medline ® (National Library of Medicine), NCBI’s PubMed, and Embase ® (Excerpta Medica Database, Elsevier), we compiled a list of 162 genes with linkage or mutations reported in human individuals with CL, and regarded them as candidate genes for human CL (Fig. 1a). We also identified 16 miRNAs that targeted these 162 genes from four miRNA-target interaction databases: miRanda, PITA, TargetScan and miRTarBase [25–28] (Additional file 1). In these networks, there are four types of regulatory interactions: TF regulation of gene expression (TF-gene), TF regulation of miRNA expression (TF-miRNA), miRNA regulation of gene expression (miRNA-gene) and miRNA regulation of TF expression (miRNA-TF). (Fig. 1b). To incorporate these regulations into miRNA-TF co-regulatory networks, we included only the miRNA- and TF-mediated FFLs (Fig. 1c and d). We combined different types of FFLs into networks and then performed functional analysis. To evaluate whether the computational analysis-driven interplays between regulators and their targets were consistent with the previous studies, we introduced a network edge validation strategy using an independent expression profiling data set (GEO accession number: GSE7759). We pinpointed important hub nodes (TFs, miRNAs, and genes) from a combined network and performed Gene Ontology (GO) gene set enrichment analysis of their targets (Fig. 1e). The GO enrichment results were consistent with previous studies and these hubs could be ideal candidates for further biological experiments. We extracted three network modules from the combined network and found the enrichment of genes involved in “the signaling pathway regulating pluripotency of stem cells” (Fig. 1f). Genes related to the Wnt pathway, a well-known pathway in the etiology of CL, were extracted and used to construct a network, in which we identified candidate diseasing-causing miRNAs (Fig. 1g). For the gene list derived from our procedures, we further performed GO and KEGG pathway enrichment analysis (Fig. 1h).

### Highly confident regulatory relationships among miRNAs, TFs and genes

Table 1 summarizes four types of potential regulatory relationships (TF-gene, TF-miRNA, miRNA-gene, and miRNA-TF) and measurement methods we used in this study.

**Table 1:** Summary of the integrated regulatory relationships among miRNAs, TFs and genes

Relationship	Number of pairs	Number of miRNAs	Number of genes	Number of TFs	Method^a^
miRNA-gene	77	9	37	/	A
miRNA-TF	21	8	/	12	A
TF-gene	671	/	126	27	B
TF-miRNA	76	17	/	22	B
Total	845	18	127	27	

^a^In the Method column, (A) regulation relationships were supported by at least two of these databases: TargetScan, miRanda, PITA and miRTarBase; (B) TRANSFAC Match™ method was used for identification of TF-target relationship

Prediction of miRNA targets has often a high false positive rate. To reduce such noises, we used the miRNA–target interactions in humans that were predicted by at least two of the four datasets: TargetScan, miRanda, PITA and miRTarBase [25–28] using stringent criteria. We obtained 77 miRNA-gene pairs and 21 miRNA-TF pairs. To obtain the regulatory relationships for each TF with its genes or miRNAs, we investigated TFs and their binding motifs in the TRANSFAC Professional database and predicted TF-gene and TF-miRNA interactions by using the Match™ program [29]. To minimize false positive results, we selected only the regulatory relationships conserved well among human, rat, and mouse [20, 23]. These data processes resulted in 671 TF-gene pairs and 76 TF-miRNA pairs (Table 1; Additional file 2).

### CL-specific miRNA-TF mediated regulatory networks with 64.8% TF-gene edges being verified

Our networks were established based on the transcriptional regulation of TFs (e.g. TF-gene, TF-miRNA) tightly coupled with the post-transcriptional regulation of miRNAs (e.g. miRNA-gene, miRNA-TF). These two types of gene regulators tend to form 3-node FFLs (each FFL has a TF, a miRNA and a joint target gene). In this study, we only considered three types of 3-node FFL motifs in CL. We named them motifs A, B, and C (Table 2). Based on the four types of regulatory relationships obtained above, a total of 128 FFLs belonged to these three types of FFL motifs (Table 2; Additional file 3).

**Table 2:** Summary of 3-node feed forward loops (FFLs)

		Number of nodes			Number of pairs			
Motif	#FFLs	TFs	miRNAs	Genes	miRNA-gene	miRNA-TF	TF-gene	TF-miRNA
A	71	12	7	26	39	/	68	17
B	50	7	6	16	30	13	31	/
C	7	2	2	6	7	2	7	2
Total	128	16	12	28	69	13	99	19

**Fig. 2:**
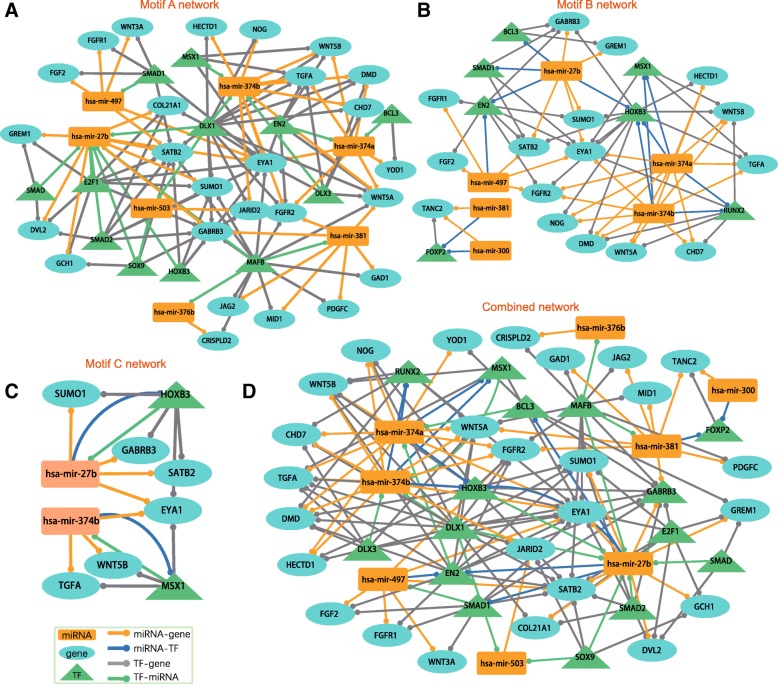
Feed-forward loop (FFL) based regulatory networks. Three types of motifs (motif A, B and C) were used to construct (**a**) Motif A network, (**b**) Motif B network, (**c**) Motif C network, and (**d**) Combined network (using all three motif types)

In motif A, the TF regulates its targeted protein-coding gene (non-TF gene) and miRNA at the transcriptional level, and the miRNA regulates its targeted protein-coding gene (non-TF) at the post-transcriptional level. In motif B, the TF regulates its targeted protein-coding gene (non-TF gene) at the transcriptional level, and the miRNA regulates both TF and protein-coding gene (non-TF gene) at the post-transcriptional level. In motif C, the regulators (TF and miRNA) mutually regulate each other, while the TF regulates the targeted protein-coding gene (non-TF gene) at the transcriptional level, and the miRNA regulates the targeted protein-coding gene (non-TF gene) at the post-transcriptional level. We constructed motif A, B, and C co-regulatory networks by merging these motif A, B, and C FFLs, respectively (Fig. 2a, b, and c). In addition, we merged all these motif FFLs to construct a combined regulatory network (Fig. 2d). The combined network consisted of 8 miRNAs, 15 TFs, 26 genes and 163 edges (Additional file 4).

To examine whether the regulatory relationships between TFs and targeted genes were reproducible, we used an independent gene expression data set (GEO accession number: GSE7759). To determine if an edge (the link between two nodes in the network) could be verified, we required that the co-expression relationship between TF and gene should be observed in the validation data set, and we used the Pearson correlation coefficient |*r*| > 0.3 to nominate the co-expression. Furthermore, the Pearson correlation *P*-values were required to be less than 0.05 with its false discovery rate (FDR) being less than 0.1. The detailed network edge validation approach was described in the Materials and methods section. Among the 88 TF-gene edges, 57 (64.8%) edges were verified by the independent data set (Additional file 5).

**Fig. 3:**
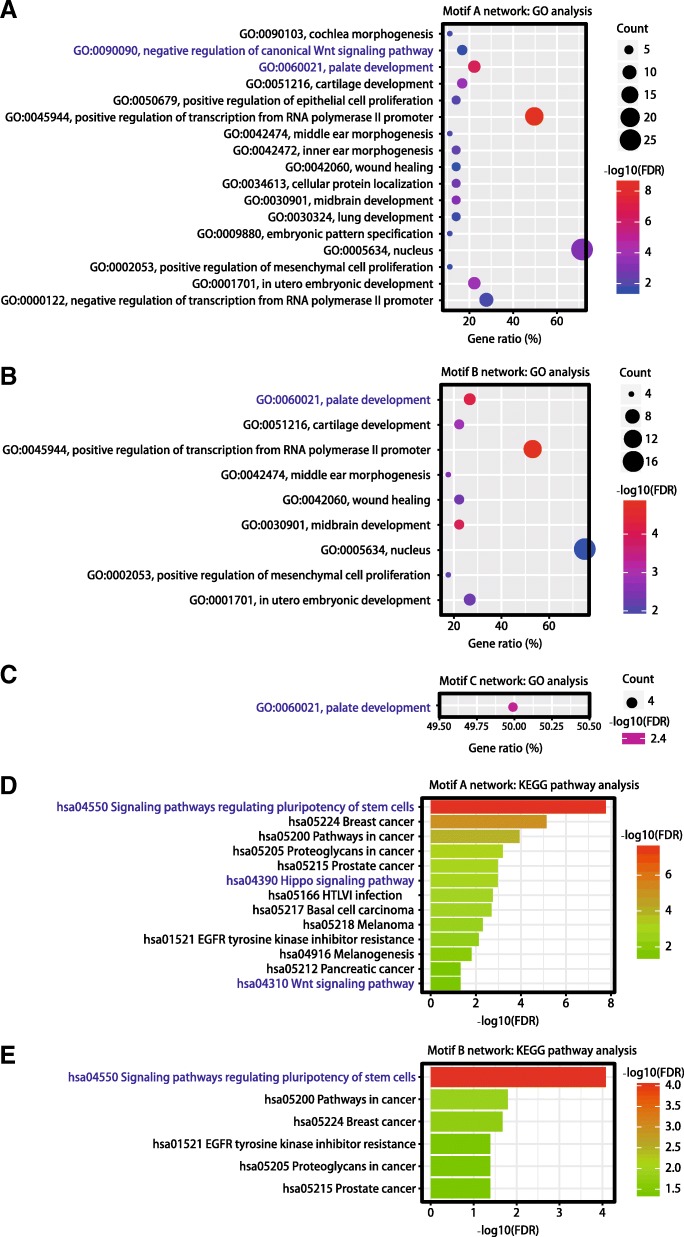
Enrichment of GO and KEGG pathways in TFs and genes in each motif network. (**a**) GO analysis of TFs and genes in motif A network. (**b**) GO analysis of TFs and genes in motif B network. (**c**) GO analysis of TFs and genes in motif C network. (**d**) KEGG pathway enrichment analysis of TFs and genes in motif A network. (**e**) KEGG pathway enrichment analysis of TFs and genes in motif B network. FDR: false discovery rate

Next, we investigated enrichment of GO and KEGG pathway annotations in these TFs and genes from each motif network. Our results were insightful into CL biological functions (Fig. 3; Additional file 6). There were 17, 9, 1 important GO terms and 13, 6, 0 key KEGG pathways in motif A, B, and C networks, respectively. Among them, “negative regulation of canonical Wnt signaling pathway (GO:0090090)”, “palate development (GO:0060021)”, “signaling pathways regulating pluripotency of stem cell (hsa04550)”, “Hippo signaling pathway (hsa04390)”, “Wnt signaling pathway (hsa04310)” have been previously reported in CL [30–35]. Interestingly, as CL occurs with cleft palate at approximately 50% of all craniofacial cleft cases, “palate development (GO:0060021)” was observed across all the three motif networks (Additional file 6).

### Prominent hub genes in regulatory networks

**Fig. 4:**
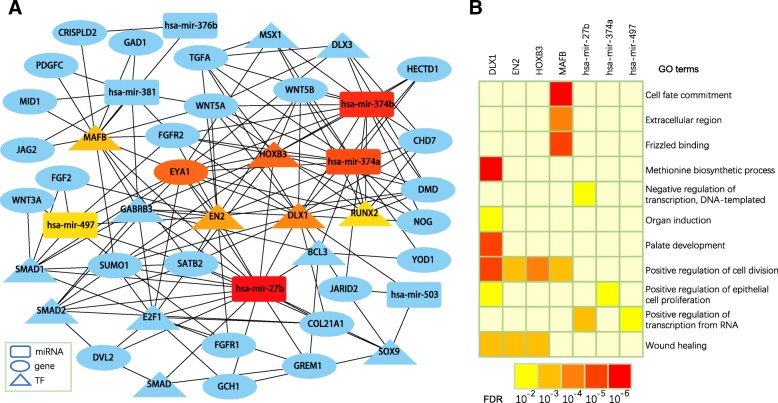
Hub nodes in the combined network and GO enrichment analysis of their targets. (**a**) Hub nodes in the combined network. Hub nodes are indicated with a color scheme from highly essential (red) to essential (yellow). (**b**) Enriched GO terms of the targeted genes of the hub miRNAs and TFs in (**a**)

Based on maximal clique centrality (MCC), a novel topological analysis method introduced in [36], we pinpointed hub nodes in the combined network using the cytoHubba plugin (version 0.1) for Cytoscape (version 3.6) [36]. We obtained ten hub nodes, including three miRNAs, five TFs and one protein-coding gene (non-TF gene) (Fig. 4a).

For each hub TF and each miRNA, we first extracted its targets and then performed GO analysis for them using the DAVID software (version 6.8) [37]. We observed a set of four hub TFs and three hub miRNAs whose targets were significantly enriched in 11 GO terms that are closely related to CL-specific biological processes (hypergeometric test *P* < 0.05, followed by the Benjamini-Hochberg procedure for multiple testing correction [38]) (Fig. 4b; Additional file 7). The results from functional enrichment of target genes implied that these hub regulators might have essential roles in CL development. Targets of four TFs (DLX1*,* EN2, HOXB3, and MAFB) were enriched in the process of positive regulation of cell division that is an important biological process in the lip development. Prior to completion of formation of the upper lips, the lateral nasal process has a climax of cell division that leave upper lips susceptible to teratogenic insults, and any perturbation in growth at this critical moment can result in malfunction of the closure mechanism [2, 39]. Furthermore, targets of three TFs (DLX1*,* EN2, and HOXB3) were significantly associated with the following biological processes: wound healing and positive regulation of transcription from RNA polymerase II promoter. These biological processes have previously reported to be associated with CL [40–42].

### Network modules revealed novel pathways involved in CL

**Fig. 5:**
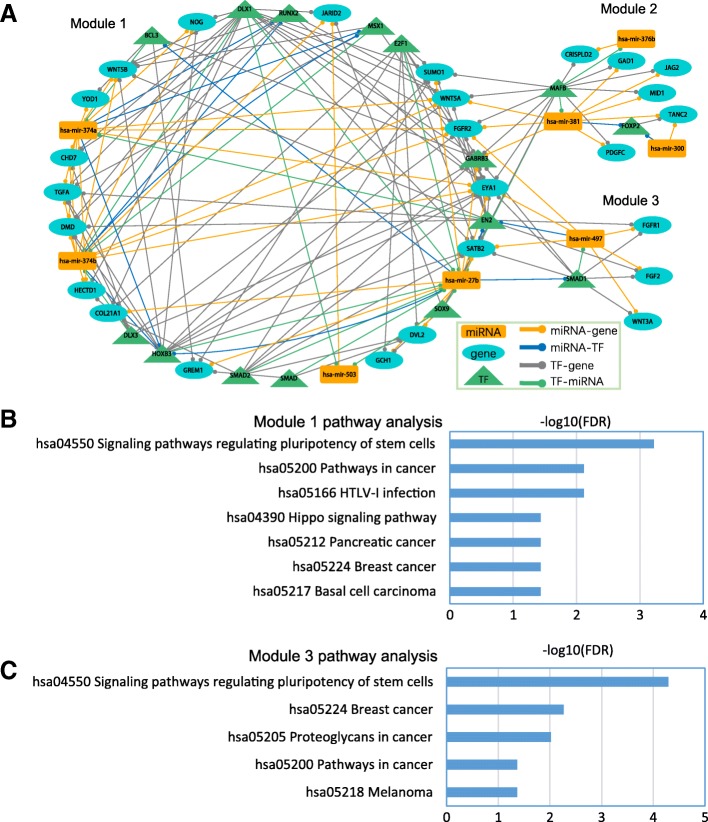
Network modules and their functional analysis. (**a**) Network modules identified from the combined network. (**b**) KEGG pathway analysis of TFs and genes in module 1. (**c**) KEGG pathway analysis of TFs and genes in module 3

The Markov clustering (MCL) algorithm [43] is a highly efficient and reliable network clustering algorithm based on simulating random walks within an interaction network. We used MCL in order to divide regulatory network into gene interaction modules. With the inflation value set to 2.0, MCL detected three modules from the combined network (Fig. 5a). Each module was composed of a set of miRNAs, TFs, and genes that were topologically adjacent. These modules were taken for further functional analysis.

We aimed to reveal critical regulations that might control the etiology of CL. Pathway analysis has been demonstrated as a helpful method to investigate the biological mechanisms implicated in the pathogenesis [44]. We explored enriched pathways in these regulatory modules. Modules 1 and 3 were enriched in several pathways (FDR < 0.05, hypergeometric test followed by Benjamini-Hochberg multiple testing correction), while module 2 was not enriched in any pathways (Additional file 8; Fig. 5a and b). This suggests that through identification of modules we can narrow down candidate genes and thus help us find critical regulations. Interestingly, both modules 1 and 3 were enriched in the signaling pathway regulating the pluripotency of stem cells. Although this pathway has not been reported to be involved in CL yet, we could find some evidence in this pathway. *SOX2* is known as one of the core genes in pluripotency and one of the transcriptional factors crucial for the reprogramming of mature cells into pluripotent stem cells [45, 46]. *SOX2* is highly correlated with *FOXE1* [47], whose congenital mutations cause cleft palate and hypothyroidism [48].

### Identification of potential disease-causing miRNAs in Wnt pathway in CL

Several studies have demonstrated that Wnt pathway is involved in non-syndromic CL [30, 49, 50]. For example, mutations in the *WNT3* gene dominate autosomal recessive tetra-amelia with CL [1, 32]. A single SNP (dbSNP ID: rs7205289) located in *pre-miR-140* contributed to the susceptibility of non-syndromic cleft palate (NSCP) by influencing the processing of *miR-140* [51]. These findings have motivated us to further analyze miRNAs in the Wnt pathway as candidate causative regulators for CL.

**Fig. 6:**
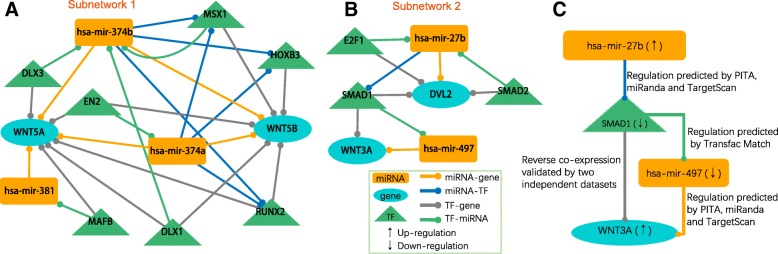
miRNAs might act as the CL regulators through Wnt pathway. (**a**) Subnetwork 1. (**b**) Subnetwork 2. (**c**) Schematic model of *hsa-mir-27b* regulation in Wnt pathway. An upregulated *hsa-mir-27b* suppresses *SMAD1* expression, which accordingly leads to an upregulation of *WNT3A* due to low level of SMAD1. ↑ represents up-regulation and ↓ represents down-regulation

Based on the KEGG pathway database [52], our miRNA-TF co-regulatory networks could recruit four genes in the Wnt pathway: three WNT family genes (*WNT3A*, *WNT5A*, and *WNT5B*) and the *DVL2* gene. We extracted two subnetworks from the miRNA-TF co-regulatory combined network by merging all the motifs that included at least one of these four genes. These subnetworks included 36 edges, five miRNAs, ten TFs and four genes (Fig. 6a and b). The subnetworks showed some interesting results. For example, *WNT3A* and *WNT5A* that appeared in these subnetworks were well-known involved in regulating upper lip fusion and mid-face development and were therefore especially strong candidates for an etiological role in CL [53, 54]. Furthermore, mutations in *MSX1* were associated with tooth agenesis and orofacial clefting in human [55].

Subnetwork 2 was composed of the core Wnt pathway gene *WNT3A* and five regulators (two miRNAs: *hsa-mir-27b* and *hsa-mir-497*; and three TF genes: *SMAD1*, *SMAD2,* and *E2F1*). Among these five regulators, we identified two miRNAs (*hsa-mir-27b* and *hsa-mir-497*) as promising CL critical hub nodes. The direction of the network edges demonstrated that *WNT3A* expression could be regulated by two miRNAs at the post-transcriptional level and regulated by *SMAD1* at the transcriptional level (Fig. 6b). Of note, *SMAD1-DVL2* and *SMAD1-WNT3A* were strongly validated by the independent validation data set (Fig. 6c). *WNT3A* expression could occur through up- or down-regulation of miRNAs that target *WNT3A*.

Previous studies have shown that *hsa-mir-27b* is upregulated in the CL region compared to normal tissues [16, 56]. Another previous study has confirmed the downregulation of *hsa-mir-497* in fibroblasts from individuals with non-syndromic isolated cleft palate [57]. In the independent validation data set, *SMAD1* and *WNT3A* were downregulated and upregulated compared to normal tissues, respectively. Although there has been no report on *hsa-mir-27b* as a CL-causing miRNA, our analysis could lead to the following putative regulation pathways (Fig. 6c): hsa-mir-27b inhibits *SMAD1* expression and then *SMAD1* inhibits *WNT3A* expression. Collectively, *hsa-mir-27b* and *hsa-mir-497* may be candidate CL miRNAs involved in the etiology of CL via the Wnt pathway.

## Discussion

During the past decade, association studies (e.g. GWAS) and candidate gene studies have identified a good number of genes or loci related to CL. The identification of candidate CL genes has traditionally been based on developmental analyses and gene expression [1]. However, with very scarce human samples available for CL gene expression assay, such studies have mostly been performed in model animals, particularly in mice and zebrafish. The assumption is that the function and phenotypic changes related to the CL genes and mutagenesis in animals can be similarly reflected in humans. With this scenario, the mutations that are associated with CL in human studies (e.g. GWAS) may be better understood through manipulation in animals or measured in early craniofacial development. Since lip development involves dynamic changes and regulation, gene regulation network based on FFLs would be a promising approach to explore the molecular regulation among those previously identified as CL candidate genes. In principle, gene regulation network assumes that many of the disease-causing genes are prone to collaboratively take effect at the network level or biological pathway rather than at an individual gene level [58].

In this study, we constructed TF and miRNA co-regulatory networks using our curated CL genes in human, aiming to find insightful regulatory motifs and networks that are related to the etiology of CL. Our framework started with the curated genes and miRNAs in human CL; this effort of candidate gene selection and curation has not been done in the dental biology field yet. We inferred confident regulatory relationships among the TFs, miRNAs and genes using a whole set of computational tools and algorithms. Based on these relationships, we focused on 3-node FFL motifs to build up CL-specific regulatory networks. This TF-miRNA-gene 3-node FFL is a power way to examine the gene regulation at both the transcription and post-transcriptional levels. However, one critical issue in such a computational approach is how to best control the false positives during the establishment of the regulatory interaction relationships. To reduce the rate of false-positive outcomes, we took the following steps. First, we selected the most recognized software, algorithms and annotated databases to predict the regulation pairs between TF-gene, TF-miRNA, miRNA-gene, and miRNA-TF. Second, we applied stringent parameters to choose miRNA-target and TF-target relationships (see Materials and methods). Third, we required the predicted pairs from more than one databases or tools. Thus, the results seemed reliable, as we verified by using an independent dataset. Overall, our framework can be applied to other complex diseases to detect regulatory relationships, important genes, critical molecular modules, and disease-causing regulators.

Among the results, the three hub miRNAs (*hsa-mir-27b*, *hsa-mir-374a,* and *hsa-mir-497*) and four hub TF genes (*DLX1*, *EN2*, *HOXB3,* and *MAFB*) were identified with relatively higher number of targets. The target genes of these regulators were involved in the CL-related biological processes. Therefore, these hub nodes may be vital candidates for further functional validation. In addition, through the analysis of network modules, we found the enrichment of genes involved in “the signaling pathway regulating pluripotency of stem cells”. Although no previous studies have revealed this important pathway in CL yet, there was indirect evidence to support the underlying association between this pathway and CL.

Another important contribution of this study is the CL-specific miRNA-TF regulatory network. To our knowledge, there is no such a regulatory network analysis in the dental biology field. To identify potential disease-causing regulators, we segmented the combined regulatory network and separated it into relatively small but functionally crucial subnetworks for specific pathways that have been previously reported in CL. We then used independent gene expression data sets and performed network topology analyses in order to identify critical miRNAs in these small subnetworks. Specifically, we took Wnt pathway as a case and found two critical miRNAs (Fig. 6c). Among them, *hsa-mir-27b* has already been reported in independent studies to be up-regulated in CL [16, 56]. Another study has shown that *hsa-mir-497* was down-regulated in CL [56]. The prior evidence that supports our network results demonstrated the effectiveness of our regulatory approach. The observation in Fig. 6 may reflect a more complicated regulation scheme in cellular system, and such scenario will not be detected by a typical canonical pathway or signaling pathway approach. Different from traditional pathway analysis, which usually focuses on protein-coding genes, our approach integrates miRNAs, TFs and genes into special regulatory pathways. Such pathways might be useful for explanation of mechanism of diseases.

There are four limitations in our study. First, to identify regulatory relationships among TFs, miRNAs and genes, we used stringent criteria in order to reduce false positives. This strategy might exclude some true positive regulations. Second, we did not determine the repression or activation relationship in each interaction pair (e.g. TF activates or represses a gene). Third, these networks do not bear scale-free topology in a typical biological network because the pairs of regulation used for network construction are more likely related. Fourth, to our best knowledge, there were no published samples which had matched miRNA and mRNA expression available for construction of large-scale regulatory networks. The FaceBase project [59] has generated both mRNA and miRNA expression data in mice, but has not included both miRNA and mRNA from the same tissue or sample yet. Such data will likely be available in the near future since genome technologies are under rapid advancement, and we will expand our analysis when such data are released.

## Conclusions

In this study, we have first identified TF-gene and miRNA-gene using our manually curated CL genes and other regulation databases. Then, we constructed comprehensive miRNA-TF mediated co-regulatory networks specific for human CL. Within these networks, we identified critical hub miRNAs, TFs and genes, which might take important roles in regulation process of CL. In addition, we found several novel pathways possibly associated with etiology of CL when we compared the results of functional analysis of different network modules. We also demonstrated that the CL-specific regulatory networks had critical disease-causing miRNAs. Moreover, we constructed a subnetwork containing human CL-related genes involved in Wnt pathway. Through network topological and functional analyses of Wnt pathway subnetwork, two critical miRNAs have been identified with the support from previous studies. This study not only unveils novel miRNAs for further experimental design but also provides further insight into regulatory mechanisms of human CL. To our knowledge, this is the first TF-miRNA mediated regulatory network in dental disease.

## Methods

### Human CL genes and related miRNAs

Genes involved in the pathology of CL were collected from three databases: Medline (Ovid), PubMed (NLM), and Embase (Ovid). After manual curation (every related paper was manually read), we obtained a list of 162 genes with mutations or association reported in individuals with CL, and considered them as candidate genes for human CL. Our bioinformatics analysis suggested that 16 miRNAs might be post-transcriptional regulators of CL genes. (Details in another study in house; Additional file 1).

### TF-mediated gene/miRNA regulation

The promoter sequences (− 1000/+ 200 bp of the transcription start site (TSS)) of human protein-coding genes and precursor miRNAs were obtained from the UCSC Table Browser (https://genome.ucsc.edu) [60] as described by Sun et al. [21]. The search of the TF binding sites was conducted using the Match™ program [61], which was available in the TRANSFAC ® Professional version (release 2016.1) [29]. A high-quality TRANSFAC ® matrix library and pre-calculated cutoffs were used to minimize false positive matches. To constrain the search, TFs containing a matrix score of > 0.95 and a core score of 1.00 were selected.

### miRNA-mediated gene/TF regulation

The candidate targeted genes of miRNAs were analyzed using bioinformatics analysis with multiple target prediction algorithms, including TargetScan (version 7.1), miRanda (August 2010 Release), PITA (version 6) and miRTarBase (Release 7.0) [25–28]. To obtain reliable miRNA-target pairs, a predicted miRNA-target pair for further analysis was supported by at least two of these public canonical miRNA-target databases.

### Validation of TF-gene regulation relationships

The mouse model is well-established to facilitate studies of the mechanism of human craniofacial development [62]. This is because mouse and human have strikingly similar craniofacial development and well conserved molecular mechanism [62–64]. To validate the TF-gene regulatory interactions in the combined network, an independent mouse gene expression data set was obtained from the Gene Expression Omnibus (GEO) [65]: accession number GSE7759 [66]. GSE7759 contains 35 samples from the developing maxillary processes that eventually form the upper lip. Their gene levels were measured using Affymetrix Mouse Genome 430 2.0 Array (Affymetrix, USA). Microarray data set was normalized with Robust Multi-Chip Averaging (RMA) [67]. TF-gene pairs with the Pearson correlation coefficient (PCC) being > 0.3 or < − 0.3 and *P*-value less than 0.05 (FDR less than 0.1) were selected (Additional file 5).

### Network and subnetwork generation, analyses, and functional evaluation

In this work, we constructed three major networks. The first network was the CL-specific miRNA-TF mediated gene regulatory network, which was generated by converging all 3-node motifs (Fig. 2d). To determine the hubs in this network, cytoHubba, a java plugin for Cytoscape software, was employed. CytoHubba provides eleven topological analysis methods. We used Maximal Clique Centrality (MCC), since MCC has a better performance on the precision of predicting essential components from network among the eleven methods.

The second one consisted of three modules generated by the MCL (Markov Clustering) algorithm [43] (Fig. 5a). The MCL algorithm is designed specifically for clustering of simple or weighted graphs. The MCL algorithm finds cluster structure in graphs by a mathematical bootstrapping procedure. The results of MCL depend on the choice of an inflation parameter (I). We applied MCL to the networks constructed with default parameters (inflation parameter = 2.0) to identify the functional clusters. Clusters with less than three nodes were removed as less biological meaning.

The third one was the subnetwork for Wnt pathway (Fig. 6a and b). By reviewing the KEGG database (http://www.genome.jp/kegg/), we found *WNT3A*, *WNT5A*, *WNT5B* and *DVL2* were involved in Wnt pathway. We extracted all the motifs which contained at least one of these four genes to construct a subnetwork, and then inferred the potential function of miRNAs in Wnt pathway.

WebGestalt [68] was used to examine the KEGG pathways that were enriched with a gene list. The significance level of pathways was set to FDR (Benjamini-Hochberg) less than 0.05. The DAVID Functional Annotation Tool was used to identify enriched Gene Ontology (GO) annotations [37].

Statistical analyses were conducted using R 3.3.1 (the R Foundation for Statistical Computing; https://www.r-project.org/). The networks were visualized using the Cytoscape software version 3.6 (http://cytoscape.org/) [69].

## Additional files

Additional file 1: **Table S1.** Human CL related 162 genes and 16 miRNAs. (XLSX 11 kb)

Additional file 2: **Table S2.** Regulatory relationships among miRNAs, TFs and genes. (XLSX 22 kb)

Additional file 3: **Table S3.** 3-node feed forward loops (FFLs). (XLSX 12 kb)

Additional file 4: **Table S4.** Regulatory relationships among miRNAs, TFs and genes in the combined network. (XLSX 13 kb)

Additional file 5: **Table S5.** Validation of TF-gene pairs using an independent gene expression data set. (XLSX 18 kb)

Additional file 6: **Table S6.** Enrichment of GO and KEGG pathway annotations of TFs and genes in each motif network. (XLSX 86 kb)

Additional file 7: **Table S7.** GO analysis of targets of hub miRNAs and TFs. (XLSX 12 kb)

Additional file 8: **Table S8.** KEGG pathway analysis of network modules. (XLSX 11 kb)

## Abbreviations

3-UTR: 3′ untranslated region
BH: the Benjamini-Hochberg procedure
CL: cleft lip
CP: cleft palate
DAVID: the database for annotation, visualization and integrated discovery
FBL: feedback loop
FDR: the false discovery rate
FFL: feed-forward loop
GO: Gene Ontology
GWA: genome wide association
KEGG: the Kyoto Encyclopedia of Genes and Genomes database
MCL: Markov clustering
miRNA: microRNA
mRNA: messenger RNA
NSCL/P: non-syndromic cleft lip with or without cleft palate
NSCP: nonsyndromic cleft palate
PCC: Pearson correlation coefficient
SNP: single nucleotide polymorphism
TF: transcription factor
TFBS: transcription factor binding site
TGFA: transforming growth factor, alpha
TSS: transcriptional start site
UCSC: the University of California Santa Cruz
